# Variants associated with Bedaquiline (BDQ) resistance identified in Rv0678 and efflux pump genes in *Mycobacterium tuberculosis* isolates from BDQ naïve TB patients in Pakistan

**DOI:** 10.1186/s12866-022-02475-4

**Published:** 2022-02-25

**Authors:** Dania Khalid Saeed, Sadia Shakoor, Safina Abdul Razzak, Zahra Hasan, Saba Faraz Sabzwari, Zahida Azizullah, Akbar Kanji, Asghar Nasir, Samreen Shafiq, Najia Karim Ghanchi, Rumina Hasan

**Affiliations:** 1 Department of Pathology and Laboratory Medicine, The Aga Khan University, Karachi, Pakistan; 2 Faculty of Infectious and Tropical Diseases, London School Hygiene and Tropical Medicine, London, UK

**Keywords:** Extensively drug resistant *Mycobacterium tuberculosis*, Bedaquiline resistance, Resistance associated variants, Whole genome sequencing

## Abstract

**Background:**

Mutations in the *Rv0678*, *pepQ* and *atpE* genes of *Mycobacterium tuberculosis* (MTB) have been reported to be associated with reduced antimycobacterial susceptibility to bedaquiline (BDQ). Resistance conferring mutations in treatment naïve MTB strains is likely to have implications for BDQ based new drug regimen that aim to shorten treatment duration. We therefore investigated the genetic basis of resistance to BDQ in MTB clinical isolates from BDQ naïve TB patients from Pakistan. In addition, mutations in genes associated with efflux pumps were investigated as an alternate mechanism of resistance.

**Methods:**

Based on convenience sampling, we studied 48 MTB clinical isolates from BDQ naïve TB patients. These isolates (from our strain bank) included 38 MDR/pre-XDR/XDR (10 BDQ resistant, 8 BDQ intermediate and 20 BDQ susceptible) and 10 pan drug susceptible MTB isolates. All strains were subjected to whole genome sequencing and genomes were analysed to identify variants in *Rv0678, pepQ, atpE, Rv1979c, mmpLS and mmpL5* and drug resistance associated efflux pump genes.

**Results:**

Of the BDQ resistant and intermediate strains 44% (8/18) had variants in *Rv0678* including; two reported mutations S63R/G, six previously unreported variants; L40F, R50Q and R107C and three frameshift mutations; G25fs, D64fs and D109fs.

Variants in efflux pumps; *Rv1273c* (G462K), *Rv0507c* (R426H) and *Rv1634c* (E198R) were found to be present in drug resistant isolates including BDQ resistant and intermediate isolates. E198R in efflux pump gene *Rv1634c* was the most frequently occurring variant in BDQ resistant and intermediate isolates (*n* = 10).

**Conclusion:**

We found RAVs in *Rv0678* to be commonly associated with BDQ resistance. Further confirmation of the role of variants in efflux pump genes in resistance is required so that they may be incorporated in genome-based diagnostics for drug resistant MTB.

**Supplementary Information:**

The online version contains supplementary material available at 10.1186/s12866-022-02475-4.

## Background

Multi- and extensively drug resistant tuberculosis (MDR/XDR TB) is difficult to treat and poses a challenging problem in the management of tuberculosis. New and re-purposed drugs have been introduced to improve outcomes of patients with drug resistant tuberculosis [1]. Bedaquiline (BDQ); a novel diaryl quinolone, has been shown [2] to be effective against both actively replicating and dormant *Mycobacterium tuberculosis* (MTB). The increased efficacy of anti-mycobacterial regimens that include BDQ as treatment for MDR TB is well illustrated [2–4]. BDQ along with delamanid (DLM) was given accelerated approval by the United States Federal Drug Authority (U.S F.D.A) for treating M/XDR TB [2], highlighting the role of BDQ in circumventing the challenge of treating drug resistant TB. As of 2018, the World Health Organization (WHO) has included BDQ as an essential drug to be used along with fluoroquinolones and linezolid for treating MDR TB [5].

Studies investigating mutations conferring resistance to BDQ, derived in vitro [6] or in clinical isolates with high minimum inhibitory concentrations (MICs) post-treatment [6, 7], have identified resistance associated variants (RAVs) arising in *atpE*, *Rv0678*, and *pepQ* genes. Genomic variants D28N and A63V in *atpE* gene in two BDQ resistant clinical isolates have also been reported [8]. Moreover, RAVs in *Rv0678* have been reported in BDQ-naïve MDR TB population [7, 9], with one study reporting 4.6% of BDQ-naïve MDR MTB isolates carrying RAVs in *Rv0678* and *pepQ* genes [9]. Worse treatment outcomes are reported in patients infected with MTB strains possessing baseline or emergent *Rv0678* variants [10, 11]. Similarly, co-occurrence of loss of function mutations in *Rv0678* and *mmpL5* in MTB isolates has also been reported [12]. These data underscore the need to continue surveillance of BDQ resistance and to establish presence of RAVs in *Rv0678* and *mmpLS-mmpL5* in baseline MDR TB isolates from patients before starting therapy. Keeping in mind the diversity of RAVs in *Rv0678* [9, 13, 14] and variations in associated MICs [9], mutation hotspots should be catalogued and characterized. Furthermore, genomic variants associated with MICs to BDQ need to be explored towards interpreting molecular results with confidence. Moreover, the recently published catalogue of resistance associated genetic variants by the WHO [15] does not categorize any of the variants in *Rv0678, Rv1979c, and mmpL5* to be associated with BDQ resistance due to the rarity of the variants reported in these genes. As more data is collected on RAVs for BDQ there will be greater information available regarding the sensitivity of these mutations in predicting a drug resistant genotype.

Efflux pumps (EPs) are recognized as contributing to resistance in MTB [16–18]. EPs in general have been shown to be to be associated with low level intrinsic drug resistance [19]. Earlier studies [18, 20] have identified SNPs in efflux pumps in XDR MTB suggesting that increased expression of SNPs may have a role in drug resistance. Resistance to BDQ is reported to arise through over-expression of the Resistance Nodulation Cell Division Super Family (RND) efflux pump *mmpLS*-*mmpL5*, linked to mutations arising in the transcriptional repressor *Rv0678* gene [21]. Presence of nonsynonymous single nucleotide polymorphisms (nsSNPs) in efflux pump genes other than *mmpLS*-*mmpL5* have not been reported in BDQ resistant MTB isolates to date [22].

Through this research, we aimed to study RAVs in BDQ naïve population of pre-XDR/XDR MTB isolates from Pakistan, using whole genome sequencing (WGS) to simultaneously identify RAVs in the known genes contributing to BDQ resistance. We further aimed to analyse genomes of BDQ resistant/ intermediate strains for variants in drug resistance associated efflux pump genes.

## Materials and methods

### Mycobacterial isolates, identification and culture

This study was conducted on MTB strains isolated from clinical specimens of BDQ naïve patients (January 2015 to June 2019) and stored as part of the Aga Khan University Hospital Mycobacteriology laboratory strain bank. Using convenience-based sampling, BDQ intermediate and resistant isolates (*n* = 18), as well as randomly selected BDQ susceptible MTB isolates; MDR/pre-XDR (*n* = 20), and pan drug susceptible MTB isolates (*n* = 10) were included in the study.

Frozen vials of MTB isolates that had been stored at -80 °C were thawed, and sub-cultured in MGIT (Becton Dickinson, USA), on Lowenstein Jensen agar slants, and on Middlebrook 7H10 with OADC (Oleic acid, albumin, catalase, dextrose) enrichment (BBL Microbiology Systems, Cockeysville, MD, USA) medium. Following sub-culture, isolates were re-confirmed to be MTB using MPT 64 Antigen test using BD MGIT TBc Identification Test kits (Cat. no 245159) and p-nitro benzoic acid test (PNB) [23].

### Drug susceptibility testing

Drug sensitivities in these isolates had been confirmed using agar proportion methods on Middlebrook 7H10 medium (BBL) for rifampicin (RIF); 1 μg/ml, isoniazid (INH); 0.2 μg/ml, ethambutol (EMB); 5 μg/ml, levofloxacin (LVX); 1 μg/ml, ethionamide (ETH); 5 μg/ml, amikacin (AMK); 2 μg/ml [5] and kanamycin (KAN); 5 μg/ml [24]. Pyrazinamide (PZA) sensitivity had been determined by the MGIT 960 method using 100 μg/ml [25]. For the 7H10 agar proportion method, critical concentration (cc) for RIF; 1 µg/ml has been revised to 0.5 µg/ml [26]. It no longer remains a valid cc for RIF. Similarly, the cc for KAN has been lowered from 5 μg/ml to 4 μg/ml [27].

Susceptibility to BDQ for MDR isolates (resistant to at least RIF and INH) was determined based on 7H10 agar dilution [28] and broth microdilution (BMD) methods [29] (Additional File 1).

MIC to BDQ, RIF, INH, ofloxacin (OFX), LVX, moxifloxacin (MXF), kanamycin (KAN), AMK, capreomycin (CAP), linezolid (LZD), CFZ and EMB were tested using frozen microsensititer plates (Thermo Fisher Scientific Inc., Waltham, MA, USA, for research purpose only), provided by Janssen Pharmaceuticals (Beerse, Belgium), in accordance with Kaniga, Cirillo et al. 2016 [29].

Based on testing by the BMD method, isolates were categorized as susceptible, intermediate or resistant to BDQ. The following cut-offs values were used for interpreting susceptibility to BDQ: isolates with MIC ≤ 0.12 µg/ml were categorized as susceptible, whereas those with an MIC ≥ 0.5 µg/ml were considered resistant. Isolates with a BDQ MIC of 0.25 µg/ml were categorized as intermediate in accordance with studies [9, 30].

The following MIC cut off values were used to determine resistance to the second-line drugs tested by BMD method: OFX (> 4.0 µg/ml), LVX(> 2.0 µg/ml), MXF(> 1.0 µg/ml), KAN(> 8.0 µg/ml), AMK(> 4.0 µg/ml), CAP(> 8.0 µg/ml), LZD(> 4.0 µg/ml), CFZ(> 0.5 µg/ml) [30].

BDQ susceptibility in MDR/pre-XDR/XDR MTB isolates with intermediate or resistant phenotypes had further been confirmed using 7H10 Agar Proportion and MGIT 960 method [29] (Additional File 1).

MDR strains were categorized as pre-XDR and XDR according to the updated definition of WHO [31]. MDR/RR-TB strains that were also resistant to any fluoroquinolone were considered as pre-XDR. While XDR-MTB strains included pre-XDR strains resistant to either or both of the group A drugs; LZD or BDQ.

For purposes of this study, drug susceptible strains (DS) were those that were susceptible to RIF, INH, EMB, PZA, fluoroquinolones, ETH, AMK, KAN, LZD, BDQ and CFZ. BDQ susceptibility in these strains (*n* = 10) was confirmed by broth microdilution method and MGIT 960. Phenotypic susceptibilities to the MDR/pre-XDR/ XDR MTB isolates (*n* = 38) were tested using broth microdilution method after revival (Additional File 1).

### DNA extraction and Whole Genome Sequencing

Genomic DNA (gDNA) was extracted from second passage of MTB grown on LJ slants using CTAB method [32]. DNA (µg/ml) was quantitated using Qubit High sensitivity assay (Invitrogen). 0.5 ng/µl of input gDNA was used for library preparation, that was carried out using Nextera XT DNA library kit (15,032,355, 15,052,163). Prepared libraries were then sequenced using the Illumina MiSeq platform (Illumina). Sequence was performed on pair end reads of 2 × 250 bp using the MiSeq reagent kit v2 [33]. Furthermore, 10 of the BDQ R/I pre-XDR/XDR MTB isolates that had low average genome coverage (< 10x) were re-sequenced using the Illumina MiniSeq platform (Illumina). MiniSeq high output reagent cartridge (15,073,286) was used.

### Bioinformatic analysis

A total of 48 samples were quality checked using FASTQC [34] and later treated with Trimmomatic v 0.32 software [35] to remove or truncate reads of low quality (parameter: LEADING: 3; TRAILING: 3; MINLEN: 36; SLIDINGWINDOW: 4:20). High quality reads were then mapped to the MTB H37Rv reference genome (GenBank accession: AL123456.3) using an in-house Bioinformatics MTB variant calling pipeline that comprises of BWA [36], Samtools [37], Picard [38] and GATK [39]. Variant calling was done using recalibration indel realignment and haplotype caller from GATK tool. Variants called from haplotype caller from GATK were filtered based on the following criteria: (1) mapping quality > 50 (-C in Samtools calling), (2) base quality/base alignment quality > 20 (-Q in Samtools calling), (3) > 10 reads (-d in Samtools filter) covering each site. Further, SNPs with a quality < 20 were removed assuming that the isolates having less than < 10 × average genome wide coverage might produce false SNP calls.

### Evaluation of genes known to confer BDQ resistance in MTB isolates

We screened for variants in genes known to confer resistance to BDQ including; *Rv0678, atpE, pepQ, mmpLS-mmpL5, Rv1979c.*

### Lineage of MTB isolates

Lineage of the MTB isolates was determined by running the Fastq files generated on TB Profiler version 3 [40].

### Evaluation of efflux pump genes

We screened for SNPs in 19 EP genes identified to have SNPs with high SIFT/Polyphen scores (S.A. Razzak and Z Hasan, unpublished data). These EP genes included; *Rv0194c, Rv1218c, Rv1217c, Rv1819c, Rv1877c, Rv0450c, Rv1634c, Rv1704c, Rv2688c, Rv0191c, Rv0507c, Rv2333c, Rv3008c, Rv3728c, Rv3756c, Rv3823c, Rv1250c, Rv1273c,* and *Rv1458c* (Additional File 2). Genomic data set of MTB isolates from our study were screened for nsSNPs in these genes in all isolates (*n* = 48).

## Results

Forty-eight MTB isolates from BDQ naïve patients were sequenced. Of these, based on BMD method, 18 MDR/pre-XDR/XDR isolates were having resistance or intermediate resistance to BDQ. Phenotypic and genotypic DST data for rifampicin (RIF), isoniazid (INH), fluoroquinolones and linezolid (LZD) for the study strains (*n* = 48) is provided in separate additional files (Additional Files 1 and 3).

The average coverage of the sequenced isolates ranged from 9.9–230.3x (Additional File 3). According to lineage characterization, 42 of the 48 MTB isolates sequenced were from Lineage 3; including all 18 BDQ resistant and intermediate isolates. 3 MTB isolates including one pan drug susceptible isolate (SS5**)** and two pre-XDR isolates (SS11, SS18) were from Lineage 4. Two isolates belonged to Lineage 2 including a pan drug susceptible MTB isolate (SS3) and an XDR isolate (S31). A pan drug susceptible isolate (SS8) belonged to Lineage 1 (Table 1).

**Table 1:** Non-synonymous single nucleotide polymorphisms (SNPs) in genes associated with bedaquiline and clofazimine resistance in clinical MTB isolates (*n* = 48)

**Resistance phenotype**			**MIC (µg/ml)**		**Variants in resistance associated genes**					
	**Study ID** **of isolates**	**Lineage**	**BDQ**	**CFZ**	***atpE*** **(1,461,045–1,461,290)**	***Rv0678*** **(778,990–779,487)**	***pepQ*** **(2,859,300–2,860,418)**	***Rv1979c*** **(2,221,719–2,223,164)**	***mmpS5*** **(778,477–778,905)**	***mmpL5*** **(775,586–778,480)**
**XDR (BDQ-R)**	S9	L3	0.5	1	-	R107C^β^	-	-	-	I948V + T794I
**XDR (BDQ-R)**	S1	L3	0.5	0.5	-	-	-	-	-	I948V + T794I
	S2				A18A	-	-	-	-	I948V + T794I
	S3				-	RAMAELQDLADVGLRALGDAPPQRSRRLREMRDLLAYMENVVSDALGRYSQRTGEDD109RGNGRTAGPGfs ^α^	-	-	-	I948V + T794I
	S4				-	SGGISTNARMLIQFGFIERLAVAGDRRTYFRLRPNAFAAGERERIRAMAELQDLADVGLRALGDAPPQRSRRLREMRDLLAYMENVVSDALGRYSQRTGEDD64SGGSAPMPGCfs^α^	-	-	-	I948V + T794I
	S5				-	L40F^β^	-	-	-	I948V + T794I
	S10					G25VCRVfs^α^	-	-	-	I948V + T794I
	S6£			0.25		R50Q^β^	-	-	-	I948V + T794I
	S7			0.25	-	-	-	-	-	I948V + T794I
	S8			0.12	-	-	-	-	-	I948V + T794I
**pre-XDR** **(BDQ-I)**	S23	L3	0.25	1	-	-		F144*		I948V + T794I
	S16	L3	0.25	0.5	-	S63R^ε^	-	-	-	I948V + T794I
	S18,				-	-	-	-	-	I948V + T794I
	S19									
	S17			0.25	-	-	-	-	-	I948V + T794I
	S20				-	S63G^ε^	-	-	-	I948V + T794I
	S22			0.12	-	-	-	-	-	I948V + T794I
**pre-XDR** **(BDQ-S)**	S31	L2	0.12	0.5	-	-	-	-	-	I948V + T794I
	S32	L3			-	-	-	-	-	I948V + T794I
	S35									
	S38									
	S40									
	S34				-	-	-	-	-	I948V + T794I
	SS15			0.12	-	-	-	-	-	I948V + T794I
	SS14			0.03	-	-	-	-	-	I948V + T794I
	S39		0.06	0.5	-	-	-	-	-	I948V + T794I
	SS18			0.06	-	-	-	-	-	I948V + T794I
	S36		0.03	0.06	-	-	-	-	-	I948V + T794I
	SS16			0.12	-	-	-	-	-	I948V + T794I
	SS19		≤ 0.008	≤ 0.015	-	-	-	-	-	I948V + T794I
**MDR (BDQ-I)**	S21	L3	0.25	1	-	-	-	-	-	I948V + T794I
**MDR (BDQ-S)**	SS16	L3	0.003	0.12	-	-	-	-	-	I948V + T794I
	SS11	L4	0.06	0.06	-	-	-	-	-	I948V
	SS18									
	SS17	L3			-	-	-	-	-	I948V + T794I
	SS20	L3	≤ 0.008	≤ 0.015	-	-	-	-	-	I948V + T794I
	SS12	L3	0.015	≤ 0.01 5	-	-	-	-	-	I948V + T794I
	SS13		0.06							
**Pan drug susceptible**	SS1	L3	0.03	0.12	-	-	-	-	-	I948V + T794I
	SS4	L2		0.03	-	-	-	-	-	I948V + T794I + D767N
	SS5	L4			-	-	-	-	-	I948V + T794I + A61A
	SS9	L3		0.06	-	-	-	-	-	I948V + T794I
	SS10									
	SS6	L3			-	-	-	-	-	I948V + T794I
	SS7									
	SS2	L3	0.06	0.12	-	-	-	-	-	I948V + T794I
	SS3	L3		0.06	-	-	R7Q	-	-	I948V + T794I + L221L
	SS8	L1			-	-	-	-	-	I948V + T794I

*BDQ* bedaquiline (MIC *R* ≥ 0.5 µg/ml, I = 0.25 µg/ml), *CFZ* clofazimine (> 0.5 µg/ml), *MDR* multidrug resistant, *XDR* extensively drug resistant, drug susceptible Mycobacterium tuberculosis (MTB) isolates as per susceptibility testing using broth microdilution method against first and second line antimycobacterials. MTB isolates were categorized into drug resistant types based on updated definitions shared by the WHO (25)

α = previously unreported frameshift mutations, β = previously unreported missense mutations, ε = previously reported missense mutation at amino acid position

WGS results revealed RAVs in *Rv0678* gene of 44% (8/18) of MTB isolates that were BDQ resistant (MIC ≥ 0.5 µg/ml) or intermediate (MIC = 0.25 µg/ml) (Table 1).

Previously unreported RAVs in *Rv0678* gene were noted in 6/10 BDQ-R MTB isolates. These included three frameshifts at amino acid positions; 25 fs in S10, 64 fs in S4 and 109 fs in S3 and three missense mutations at amino acid positions; L40F in S5, R50Q in S6 and R107C in S9 (Table 1). Additionally, previously reported variants at amino acid position 63 [7] were observed in two BDQ intermediate isolates; S63R in S16 and S63G in S20. Variants in *Rv0678* were not found in any of the BDQ susceptible MTB isolates tested. A silent mutation A18A in the *atpE* gene of XDR-MTB isolate S2 was also identified, as was a missense mutation; R7Q in the *pepQ* gene of pan drug susceptible MTB isolate SS3 (Table 1).

CFZ resistance was identified in 1/10 BDQ resistant and 2/8 BDQ intermediate strains (Additional File 1). Variant in the *Rv0678* gene were found in isolate with resistance to both CFZ and BDQ (Table 1).

**Fig. 1:**
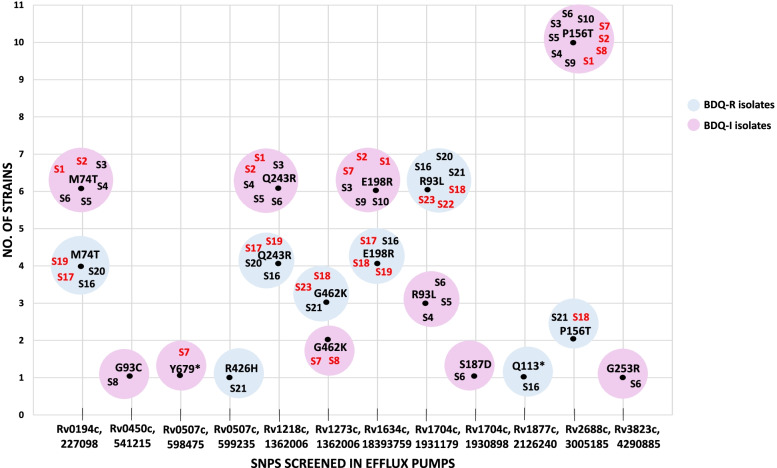
Single nucleotide polymorphisms (SNPs) found in efflux pump genes (*n* = 10) screened in *Mycobacterium tuberculosis* isolates having phenotypic resistance or raised minimum inhibitory concentration (MICs) to BDQ. The circles show MTB isolates (S1-S10, S20-S23) with SNPs identified in their efflux pump genes. Pink circles include BDQ resistant (MIC ≥ 0.5 µg/ml) isolates. Blue circles include susceptible isolates with raised BDQ MICs (MIC = 0.25 µg/ml). Red font depicts strains without genomic variants in rv0678 gene. SNPs in efflux pump genes M74T (*Rv0194c*), R426H (*Rv0507c*), Q243R (*Rv1218c*), G462K (*Rv1273c*), E198R (*Rv1634c*), P156T (*Rv2688c*) had a high Polyphen/SIFT score (SAR/ZH unpublished literature)

Variants in selected genes coding for efflux pumps (EPs) reported to play an important role in acquisition of drug resistance in MTB isolates (Additional File 2) (unpublished data) were studied. Presence of mutations in genes coding for EP in strains with full and intermediate resistance to BDQ was explored. nsSNPs were identified in 10 out of the 20 EP genes screened in these isolates including: *Rv0194c*, *Rv0450c*, *Rv0507c*, *Rv1218c*, *Rv1273c*, *Rv1634c*, *Rv1704c*, *Rv1877c*, *Rv2688c*, *Rv3823c*. SNP; P156T was found in *Rv2688c* of all BDQ resistant isolates and in 25% of BDQ intermediate isolates. Most BDQ resistant or intermediate isolates had mutations in; *Rv1704c* (*n* = 9, R93L), *Rv1218c* (*n* = 10, Q243R), *Rv1634c* (*n* = 10, E198R), *Rv0194c* (*n* = 10, M74T) (Fig. 1). Variants in genes encoding for efflux pumps; *Rv0194c* (M74T), *Rv1218c* (Q243R), *Rv1704c* (R93L), *Rv2688c* (P156T) were found in both drug susceptible and resistant MTB isolates. Variants in EP genes; *Rv0507c* (R426H), *Rv1273c* (G462K), *Rv1634c* (E198R), were detected only in MDR/pre-XDR/XDR MTB (including BDQ intermediate and resistant) isolates (Additional File 4). Amongst these nsSNP; E198R in *Rv1634c* was found to be occurring more frequently in BDQ resistant and intermediate isolates (*n* = 10). Low frequency occurring nSNPs were also found to be present in BDQ resistant and intermediate isolates. these included: Q113* in *Rv1877c* (*n* = 1), G93C in *Rv0450c* (*n* = 1), S187D in *Rv1704c* (*n* = 1), G253R in *Rv3823c* (*n* = 1), Y679* in *Rv0507c* (*n* = 2). R93L was the only previously reported lineage specific neutral mutation [41] identified in *Rv1704c* (Fig. 1).

SNPs in efflux pump genes were observed in isolates with intermediate resistance (*n* = 5) or resistance (*n* = 4) to BDQ that did not have any variants in *Rv0678*. Most frequently observed combination of EP genes with SNPs included *Rv1634c* (E198R), and *Rv2688c* (P156T) in BDQ resistant isolates (S1, S2, S7), and *Rv01273c* (G462K) and *Rv1704c* (R93L) in isolates with intermediate susceptibilities to BDQ (S18, S21, S23) (Fig. 1).

## Discussion

The inclusion of BDQ and DLM for treating MDR TB has allowed development of effective standardized short-course (SSC) regimens [42, 43]. However, reports of variants in genes conferring BDQ resistance, in M/XDR from BDQ naïve TB population are reported [7, 9]. These variants can contribute to resistance or reduced susceptibility to BDQ leading to MDR TB patients receiving sub-optimal treatment resulting in poorer outcomes and spread of resistance in the population [44].

The strains studied belonged primarily to Lineage 3 (87.5%; 42/48) but strains from Lineages 1, 2 and 4 were also present. The predominance of Lineage 3 is reflective of the molecular epidemiology of MTB in Pakistan [45]. RAVs in *Rv0678* have been reported at diverse amino acid positions including: V1A, C4R, S63R, L117R, L136P, R172W and R135G [15, 46], among BDQ resistant MTB clinical strains. Six out of the eight individually occurring variants in the *Rv0678* gene of isolates showing phenotypic BDQ resistance and raised MICs to BDQ were found to be novel. The phenotypic resistance to BDQ for isolates with variants at amino acid positions 109 and 63 differed between the clinical isolates from this study as compared to previously published studies [7, 11]. It has been recognised that SNPs at the same amino acid position could lead to variable resistance to BDQ depending on the effect of the amino acid substituted on disrupting protein function, and whether these variants are present singly or with multiple SNPs [47]. Guidelines to delineate clinical interpretation of variants in *Rv0678, Rv1979c and mmpLS-mmpL5* and its subsequent incorporation into treatment strategies are required. Studies coupling treatment outcome data with phenotypic and genotypic testing results would provide stronger evidence to interpret the clinical significance of these mutations.

Further, it is important to classify the intermediate category for BDQ resistance. As presented by Ismail et al. 2018 [9], patients (*n* = 8) with isolates having intermediate resistance (MIC = 0.25 µg/ml) were culture positive at six months of therapy. Additionally, the presence of intermediate resistance to BDQ raises concerns whether susceptibility testing at a single BDQ concentration, as in case of the MGIT 960 method, would limit understanding of the clinical challenges these phenotypes may present in context of successfully treating MDR TB. Methods such as microbroth dilution that provide a wider range of drug concentrations tested should be standardized for routine DST to capture raised MICs and intermediate resistance.

Due to the cost of fitness on MTB in vivo [46], mutations in *atp E* occur at a low frequency in BDQ resistant clinical MTB isolates [48]. While our study reports a synonymous mutation A18 in S2-a BDQ resistant XDR isolate, it is unlikely to be contributing to BDQ resistance as it is silent and does not translate into an amino acid substitution.

Variants in *Rv1979c* have been previously reported in ≤ 1% of BDQ resistant MTB isolates with no prior exposure to the drug [9]. However, association of phenotypic BDQ resistance with SNPs in *Rv1979c* is indeterminate [9, 49]. A unique SNP (F144*) in *Rv1979c* gene was also seen in one of our strains (S21) that had intermediate resistance to BDQ raising the possibility that such mutation maybe contributing to resistance in these strains.

We were unable to detect RAVs in the *Rv0678* gene of 55.55% (10 /18) of BDQ resistant and intermediate MTB isolates sequenced. These finding are supported by previous studies [49–51], suggesting that alternate mechanisms of resistance contributing to BDQ resistance need to be explored.

Our study shows presence of genomic variants in the RND efflux pump; *mmpL5* (*rv0676*)-recognised as the target of the Rv0678 repressor protein in both BDQ susceptible and resistant isolates [6]. Published studies report an association of SNPs (V344L) in *mmpL5* of a naïve BDQ resistant isolate with decreased susceptibility to antimycobacterial drugs [52]. Variants in *mmpL5* could also lead to a loss of function in the efflux pump subunit; thus increasing BDQ susceptibility. This is an important consideration when for instance *Rv0678* RAVs co-occur with putative loss-of-function mutations as reported [12]. However, *mmpL5* mutations; I948V and T794I reported here are phylogenetic variants [41].

NsSNPs were detected in four of twenty EP genes screened in our pre-XDR/XDR MTB isolates including BDQ resistant and intermediate isolates. Literature supports the role of SNPs in EP genes in contributing towards low level resistance [53, 54]. Efflux pumps; *Rv0450c (mmpL4)*, *Rv1273c*, *Rv0507c (mmpL2), and Rv1634c*, identified to have variants in MDR/pre-XDR/XDR MTB isolates including BDQ intermediate and resistant isolates have also previously been documented [20, 55] to contribute to multi drug resistance. *MmpL2* and *mmpL4* gene expression is controlled by *Rv0678* transcriptional repressor protein [56]. It is therefore possible to hypothesize that nsSNPs in *mmpL2* and *mmpL4* EP genes may impair binding of *Rv0678* leading to an over-expression of these pumps, contributing towards BDQ resistance. As variants in these efflux pumps were found exclusively in drug resistant MTB isolates, including BDQ resistant and intermediate strains, they should be studied in MTB clinical isolates for their potential role as diagnostic markers of multidrug resistance. However, transcriptomic studies of MTB drug resistance isolates are required to provide a clearer understanding of the association between genetic mutations and alterations in gene expression towards development of drug resistance [57].

Variants in BDQ naïve XDR MTB isolates reported in our study, as well as previously described acquisition of BDQ resistance during therapy [58] support genotypic and phenotypic DST at baseline and during therapy to ensure appropriate management of TB cases. The absence, amongst phenotypically BDQ resistant/intermediate isolates of variants in genes known to be associated with BDQ resistance, indicates that currently both genotypic and phenotypic DST methods are required to be used in tandem for the detection of BDQ resistance in MTB. In the context of resource-limited settings including Pakistan; implementation of WGS as a diagnostic tool is likely to present technical, logistic as well as financial challenges that need to be considered at a programmatic level [59]. However, the benefits of using WGS as a companion diagnostic to phenotypic DST as part of TB management and control cannot be ignored [60, 61]. Therefore, investment in human and infrastructural resources must be made to strengthen lab phenotypic and genotypic DST capacity.

One of the limitations of this study is that the bioinformatic pipeline used was not developed to screen for low frequency SNPs that could accurately indicate the presence of heteroresistance MTB population [62].

## Conclusion

Previously unreported variants in *Rv0678* gene of BDQ naïve MTB isolates in our strains coupled with the presence of genomic variants in EP genes in BDQ resistant and intermediate pre-XDR/XDR MTB isolates indicate the need to include genomic analysis in studying BDQ resistant strains. Our data further highlight the need to explore the role of EPs as an alternate mechanism of resistance towards including them as part of molecular screening of antimycobacterial drug resistance.

## Supplementary Information

**Additional file 1.**

**Additional file 2.**

**Additional file 3.**

**Additional file 4.**

## Data Availability

The dataset supporting the conclusions of this article is available in the SRA. The accession number for Fastq files submitted in the SRA database is: PRJNA717333 [63].

## Abbreviations

TB: Tuberculosis
MDR: Multi drug resistant
XDR: Extensively drug resistant
RR: Rifampin resistant
BDQ: Bedaquiline
MTB: *Mycobacterium tuberculosis*
DLM: Delaminid
US FDA: United States Federal Drug Authority
WHO: World Health Organization
RAVs: Resistance Associated Variants
MICs: Minimum Inhibitory Concentrations
CFZ: Clofazamine
EP: Efflux pumps
RND: Resistance Nodulation Cell Division Super Family
nsSNPs: Nonsynonymous single nucleotide polymorphisms
RIF: Rifampicin
INH: Isoniazid
EMB: Ethambutol
LVX: Levofloxacin
ETH: Ethionamide
AMK: Amikacin
KAN: Kanamycin
PZA: Pyrazinamide
CC: Critical concentration
BMD: Broth Micro Dilution
OFX: Ofloxacin
MXF: Moxifloxacin
KAN: Kanamycin
CAP: Capreomycin
LZD: Linezolid
SM: Streptomycin
