# Supplementary material for: Variants associated with Bedaquiline (BDQ) resistance identified in Rv0678 and efflux pump genes in Mycobacterium tuberculosis isolates from BDQ naïve TB patients in Pakistan
Source: BMC Microbiol. 2022 Feb 25;22:62. doi: 10.1186/s12866-022-02475-4 (PMC8876534; doi:10.1186/s12866-022-02475-4)
Supplement: Supplementary file 2 — Additional file 2. [file 12866_2022_2475_MOESM2_ESM.docx]

**Additional File 2. Efflux pump genes (n=19) with High Sift/Polyphen Indices**

| **EP genes** | **Description of EP encoded by genes** | **Anti-tuberculous drug substrate** | **Ref** |
| --- | --- | --- | --- |
| *Rv0194c* | Probable transmembrane multidrug efflux pump, ABC Transporter | INH, STP | [1, 2, 3] |
| *Rv1218c* | Probable tetronasin-transport ATP-binding protein ABC transporter | INH, RIF | [1] |
| *Rv1217c* | ABC efflux pump | - | [4, 1] |
| *Rv1819c* | Probable drug-transport transmembrane ATP-binding protein ABC transporter BacA | INH, RIF | [1, 5] |
| *Rv1877c* | Probable conserved integral membrane protein, major facilitator superfamily (MFS) | STP | [1, 6] |
| *Rv0450c* | mmpL4, conserved transmembrane transport protein, RND | RIF | [1, 6] |
| *Rv1634c* | Possible drug efflux membrane protein | INH | [2] |
| *Rv1704c* | cycA Probable D-serine/alanine/glycine transporter protein | Cycloserine | [1] |
| *Rv2688c* | efpA Possible integral membrane efflux protein | STP, CIP | [1, 6] |
| *Rv0191c* | Probable conserved integral membrane protein | PZA | [7] |
| *Rv0507c* | mmpL2, Probable conserved transmembrane transport protein, RND | INH | [8] |
| *Rv2333c* | Integral membrane drug efflux protein Stp | TET | [9, 10] |
| *Rv3008c* | Hypothetical protein | PZA | [8] |
| *Rv3728c* | Probable conserved two-domain membrane protein | INH, EMB | [6, 10] |
| *Rv3756c* | Possible osmoprotectant (glycine betaine/carnitine/choline/L-proline) transport integral membrane protein ABC transporter ProZ | PZA | [8] |
| *Rv3823c* | mmpL8, Conserved integral membrane transport protein MmpL8, RND | INH | [2] |
| *Rv1250c* | Probable drug-transport integral membrane protein | INH | [2] |
| *Rv1273c* | Probable drugs-transport transmembrane ATP-binding protein ABC transporter | INH | [1] |
| *Rv1458c* | Probable unidentified antibiotic-transport ATP-binding protein ABC transporter | INH/ RIF/EMB/STP | [11] |

**Table legend**: EP= efflux pump, ATP= Adenosine triphosphate, ABC = ATP-binding cassette transporter proteins, MFS= major facilitator superfamily, RND= resistance nodulation cell division superfamily, INH= isoniazid, RIF= rifampicin, EMB= ethambutol, STP= streptomycin, CIP= ciprofloxacin, BDQ= bedaquiline.

References

1. Jabbar A, Phelan JE, de Sessions PF, Khan TA, Rahman H, Khan SN, et al. Whole genome sequencing of drug resistant Mycobacterium tuberculosis isolates from a high burden tuberculosis region of North West Pakistan. Sci Rep. 2019; doi:10.1038/s41598-019-51562-6.

2. Narang A, Giri A, Gupta S, Garima K, Bose M, Varma-Basil M. Contribution of putative efflux pump genes to isoniazid resistance in clinical isolates of Mycobacterium tuberculosis. Int J Mycobacteriol. 2017; doi: 10.4103/ijmy.ijmy_26_17.

3. Farhat, M., Shapiro, B., Kieser, K. et al. Genomic analysis identifies targets of convergent positive selection in drug-resistant Mycobacterium tuberculosis. Nat Genet 45, 1183–1189 (2013). <https://doi.org/10.1038/ng.2747>.

4. Kanji A, Hasan R, Ali A, Zaver A, Zhang Y, Imtiaz K, et al*.* Single nucleotide polymorphisms in efflux pumps genes in extensively drug resistant Mycobacterium tuberculosis isolates from Pakistan. Tuberculosis (Edinb)*.* 2017;107:20-30.

5. Ghajavand H, Kargarpour MK, Khanipour S, Dizaji SP, Masoumi M, Jamnani FR, et al. Scrutinizing the drug resistance mechanism of multi- and extensively-drug resistant Mycobacterium tuberculosis: mutations versus efflux pumps. Antimicrob Resist Infect Control. 2019; doi: 10.1186/s13756-019-0516-4.

6. Gupta AK, Katoch VM, Chauhan DS, Sharma R, Singh M,Venkatesan K, et al. Microarray Analysis of Efflux Pump Genes in Multidrug-Resistant Mycobacterium tuberculosis During Stress Induced by Common Anti-Tuberculous Drugs. Micro. Drug Resis. 2010; doi.org:10.1089/mdr.2009.0054.

7. Zhang Y, Zhang J, Cui P, Zhang Y, Zhang W. Identification of Novel Efflux Proteins Rv0191, Rv3756c, Rv3008, and Rv1667c Involved in Pyrazinamide Resistance in Mycobacterium tuberculosis. Antimicrob Agents Chemother. 2017; doi: 10.1128/AAC.00940-17.

8. Kuan CS, Chan CL, Yew SM, Toh YF, Khoo J-S, Chong J, et al. Genome Analysis of the First Extensively Drug-Resistant (XDR) Mycobacterium tuberculosis in Malaysia Provides Insights into the Genetic Basis of Its Biology and Drug Resistance. PLOS ONE*.* 2015; doi:10.1371/journal.pone.0131694.

9. Calgin MK, Sahin F, Turegun B, Gerceker D, Atasever M, Koksal D, Karasartova D, Kiyan M. 2013. Expression analysis of efflux pump genes among drug-susceptible and multidrug-resistant Mycobacterium tuberculosis clinical isolates and reference strains. Diagn. Microbiol. Infect. Dis. 76:291–297. 10.1016/j.diagmicrobio.2013.02.033

10. De Rossi, E., P. Arrigo, M. Bellinzoni, P. A. Silva, C. Martin, J. A. Ainsa, P. Guglierame, and G. Riccardi. 2002. The multidrug transporters belonging to major facilitator superfamily in Mycobacterium tuberculosis. Mol. Med. 8:714-724.

11. Hao P, Shi-Liang Z, Ju L, et al. The role of ABC efflux pump, Rv1456c-Rv1457c-Rv1458c, from Mycobacterium tuberculosis clinical isolates in China. Folia Microbiol 56, 549–553 (2011). https://doi.org/10.1007/s12223-011-0080-7
